# White matter microstructural changes are related to cognitive dysfunction in essential tremor

**DOI:** 10.1038/s41598-017-02596-1

**Published:** 2017-06-07

**Authors:** Julián Benito-León, Virginia Mato-Abad, Elan D. Louis, Juan Antonio Hernández-Tamames, Juan Álvarez-Linera, Félix Bermejo-Pareja, Ángela Domingo-Santos, Luis Collado, Juan Pablo Romero

**Affiliations:** 1 Department of Neurology, University Hospital “12 de Octubre”, Madrid, Spain; 2 Centro de Investigación Biomédica en Red sobre Enfermedades Neurodegenerativas (CIBERNED), Madrid, Spain; 3 Department of Medicine, Faculty of Medicine, Complutense University, Madrid, Spain; 4 Neuroimaging Laboratory, Center for Biomedical Technology, Rey Juan Carlos University, Móstoles Madrid, Spain; 5 Department of Neurology, Yale School of Medicine, Yale University, New Haven, CT USA; 6 Department of Chronic Disease Epidemiology, Yale School of Public Health, Yale University, New Haven, CT USA; 7 Center for Neuroepidemiology and Clinical Neurological Research, Yale School of Medicine, Yale University, New Haven, CT USA; 8 Department of Radiology, Hospital Ruber International, Madrid, Spain; 9 Faculty of Biosanitary Sciences, Francisco de Vitoria University, Pozuelo de Alarcón, Madrid, Spain; 10 Brain Damage Service, Hospital Beata Maria Ana, Madrid, Spain; 11 Clinical Research Unit, University Hospital, “12 de Octubre”, Madrid, Spain

## Abstract

Diffusion tensor imaging (DTI) studies have detected white matter microstructural changes in essential tremor (ET). However, it is still unclear whether these changes are related to cognitive deficits, which have been described in ET patients. DTI-derived fractional anisotropy, mean diffusivity (MD), axial diffusivity (AD), and radial diffusivity measures were compared between 23 ET patients and 23 age-, gender-, and education-matched healthy individuals, using whole-brain tract-based spatial statistics. Correlations of white matter changes with scores obtained from a detailed neuropsychological assessment were subsequently examined. ET patients demonstrated increases in MD in the bilateral posterior corona radiata, bilateral superior longitudinal fasciculus, bilateral fornix (cres)/stria terminalis, genu and splenium of the corpus callosum, bilateral anterior and posterior limbs of internal capsule, bilateral retrolenticular region part of internal capsule, and left posterior thalamic radiation. Except for the genu of the corpus callosum, an increase in AD values was also found in these same tracts. Furthermore, increased MD and AD values in different white matter areas was negatively correlated with performance on language and verbal memory and positively with visuospatial ability. These correlations suggest that white matter changes might be involved in the pathogenesis of cognitive deficits in ET.

## Introduction

The emerging view of essential tremor (ET), one of the most common adult movement disorders^1^, is that it might be a family of diseases, unified by the presence of kinetic tremor, and further characterized by etiological, clinical and pathological heterogeneity^2–4^. The biological mechanisms that underlie ET are not entirely clear although there is considerable evidence to support neurodegenerative mechanisms^5^. Aside from motor manifestations, ET is also associated with a number of non-motor manifestations, including depressive symptoms^6^, changes in sleep patterns^7^, and hearing impairment^8^. In addition to these non-motor features, ET patients show mild cognitive deficits, mainly in attention and frontal executive functions, verbal memory and visuospatial processes, which might be explained by frontal cortical or frontal cortical–cerebellar pathway dysfunction^9–15^. Furthermore, cognitive deficits in ET might be not static and appear to progress at a faster rate than in normal elders^12^. In particular, individuals ET (esp. late onset ET) appear to have an increased prevalence of mild cognitive impairment and dementia^16,17^ and to have a higher risk of incident dementia^18^.

The pathogenesis of the cognitive deficits in ET is currently unknown. However, in the last years, research into the pathogenesis of cognitive dysfunction associated with ET has made some progress. Specifically, functional neuroimaging studies have detected aberrant connectivity in resting-state networks involved in cognitive processes^19,20^. Results from voxel-based morphometry studies are partly in agreement with these findings, with studies revealing widespread areas of atrophy in both the cerebellum and cerebral hemispheres (frontal and parietal lobes, insula, and cingulum)^21–23^. In particular, a recent study has shown that in cognitively impaired ET patients, executive function along with working memory correlate with gray matter volume values in the right medial frontal gyrus, anterior cingulate cortex, inferior parietal lobe, left insula, and right lobe of the posterior cerebellum^21^. Furthermore, the majority of the diffusion tensor imaging (DTI) studies have detected white-matter alterations in ET in the frontoparietal regions and cerebellum^24–27^; indeed, in the cerebello-frontal networks, which are known to play an important role in higher level cognitive functions^28^.

DTI is a MRI technique that is used to map and characterize the three-dimensional diffusion of water as a function of spatial location^29^. Several DTI parameters are used to assess diffusion and, indirectly, fiber tract microstructure. Both fractional anisotropy (FA), which measures the anisotropic diffusion of water molecules, and mean diffusivity (MD), which measures the average diffusion in the x, y, and z directions, are the most important measures of diffusion^29^. In addition, radial diffusivity (RD) describes the diffusion perpendicular to axons and axial diffusivity (AD) describes the diffusion parallel to the axon.

Despite correlations between cognition and white-matter microstructural changes that have been found in healthy individuals^30^, patients with Parkinson’s disease^31^ and patients with Huntington’s disease^32^, this relationship has not been the focus of much investigation in ET. To the best of our knowledge, only one study has examined white matter changes relative to cognitive dysfunction in ET, identifying a correlation between white matter changes in various brain regions with abnormal neuropsychological test scores only in cognitively impaired ET patients^33^. Specifically, MD, RD, and AD values were correlated with various neuropsychological test scores, mainly those measuring executive function, visuospatial function, and visual-verbal memories^33^. Nevertheless, there was no statistically significant difference in any of the diffusion measures for comparisons between cognitively unimpaired ET patients and the healthy control (HC) group^33^; as such, the meaning of this relationship is unclear.

In the current study, we used tract-based spatial statistics (TBSS) to assess white matter microstructural changes in ET patients compared with HC. Correlations of white matter changes with scores obtained from a detailed neuropsychological assessment covering the domains of attention, executive function, verbal memory, visual memory, visuospatial ability, and language, were subsequently examined.

## Methods

### Ethical aspects

All the participants included in the study gave their written informed consent after full explanation of the procedure. The study, which was conducted in accordance with the principles of the Helsinki declaration of 1975, was approved by the ethical standards committee on human experimentation at the University Hospital “12 de Octubre” (Madrid). Written (signed) informed consent was obtained from all enrollees.

### Participants

ET patients were consecutively recruited from October 2012 to July 2013 from the outpatient neurology clinics of the University Hospital “12 de Octubre” in Madrid (Spain) after obtaining an appropriate informed consent. Patients with history of dementia, stroke, epilepsy, head injury or serious medical illness were excluded. Furthermore, based on a detailed clinical mental status examination, we excluded patients with Diagnostic and Statistical Manual of Mental Disorders (DSM)–IV criteria for dementia^34^.

Two neurologists with expertise in movement disorders (J.B.-L. and J.P.R.), who were blinded to the MRI results, examined the patients and used the Fahn-Tolosa-Marìn tremor rating scale to assign a total tremor score (range = 0–144)^35^. Diagnoses of ET were assigned by the 2 neurologists (JB-L and JPR) using the Consensus Statement on Tremor by the Movement Disorder Society^36^. Furthermore, all ET patients had a normal [(123) I]FP-CIT single photon emission computed tomography scan. All eligible ET patients underwent a detailed videotaped neurological examination. Each videotape was reviewed by a senior neurologist specializing in movement disorders (E.D.L.) who re-assessed ET diagnosis using the Consensus Statement on Tremor by the Movement Disorder Society^36^. The ET patients were also followed at regular intervals (3 months, 6 months, or 12 months, based on clinical need) after the MRI procedure, and their clinical assessment, described above, was repeated. The mean duration of follow-up after the MRI procedure was 2.5 years (median = 2.7 years; range = 1.8–2.8 years).

HCs were recruited either from relatives or friends of the health professionals working at the University Hospital “12 de Octubre” of Madrid (Spain) or among the relatives of patients who came to the neurological clinics for reasons other than ET (e.g., headache, dizziness). None reported having a first-degree or second-degree relative with ET. Each control was examined by two neurologists (JB-L and JPR), who were blinded to the MRI results, to further rule out any neurological or other serious conditions, including movement disorders, dementia, stroke, epilepsy, or head injury.

### Neuropsychological testing

All participants underwent a detailed neuropsychological assessment covering the domains of attention, executive function, verbal memory, visual memory, visuospatial ability, and language. Testing was performed by a trained neuropsychologist (V. P., see acknowledgments) using standardized procedures who was blinded to the clinical diagnosis as well as the MRI results. These tests have previously been described^20^. Depressive symptoms severity was measured by the original 17-item version of the Hamilton Depression Rating Scale^37^.

In ET patients, each raw score was transformed into a standardized Z score based on the mean and standard deviation (SD) calculated from the HCs, according to the formula: Z = (raw score – mean score)/SD. Within each domain, Z scores were averaged to yield six composite scores that assessed attention, executive function, verbal memory, visual memory, visuospatial ability, and language, respectively, and that were used in subsequent correlation analyses. Individual cognitive measures were grouped into the following cognitive domains^20^:

1. Attention: Direct digit span and Coding-digit symbol subtests from the Wechsler Adult Intelligence Scale-Third Edition (WAIS-III).
2. Executive function: Stroop color–word trial, Frontal assessment battery, WAIS-III similarities subtest, Indirect digit span test from the WAIS-III, and Controlled oral word association test.
3. Verbal memory: Wechsler Memory Scale-Third Edition Word list.
4. Visual memory: Brief visuospatial memory test-revised.
5. Visuospatial ability: Benton judgment of line orientation test and Hooper visual organization test.
6. Language: Boston naming test and total number of animals as possible in one minute.

### MRI data and acquisition and analysis

As the possible effects of long-term anti-tremor medications on white matter integrity in patients with ET is unknown, we decided that all patients continued taking medication for their disease - propranolol and/or primidone during the MRI procedures.

Patients and controls were positioned in the scanner and were told to relax with their eyes closed. They were immobilized with a custom-fit blue bag vacuum mold (Medical Intelligence, Inc.) to prevent motion artifacts. Earplugs and noise-reduction headphones were used to attenuate scanner noise.

MRI scans were acquired on a General Electric Signa 3 T MRI Scanner (General Electric Healthcare, Waukesha, WI) using an 8-channel phased array coil. The diffusion-weighted image (DWI) protocol acquisition consisted of 3 images without diffusion gradients (b = 0 s/mm2) and 45 images measured with 45 directions (b = 1000 s/mm2) isotropically distributed in space. Additional parameters of the acquisition were: TE = 89 ms, TR = 10.100 ms, flip-angle = 90, slice thickness = 2.6 mm (no gap), resolution = 2.6042 × 2.6042 × 2.6 mm, FOV = 250 mm and axial acquisition.

DWI were pre-processed using FMRIB’s Diffusion Toolbox (FDT, http://fsl.fmrib.ox.ac.uk/fsl/fslwiki/FDT/), part of FMRIB Software Library (FSL)^38,39^. Pre-processing consisted of correction for eddy current distortion and head motion using the EDDYCORRECT function and field map correction using FUGUE and PRELUDE functions. Non-brain tissue from the average b0 image was removed using the FMRIB’s Brain Extraction Toolbox, BET. The brain mask was applied to the rest of the diffusion-weighted images. Next, the diffusion tensor was estimated for each voxel using the DTIFIT function via linear regression to derive FA, MD, AD and RD maps.

Subsequently, the TBSS package was used to perform voxel-wise analyses of whole-brain white matter measures (http://www.fmrib.ox.ac.uk/fsl/tbss/index.html)^40^. Briefly, individual FA images underwent nonlinear registration to the FMRIB58_FA template space and were averaged to create a mean FA image. This was then thinned to create a white matter tract “skeleton” using the default FA threshold of 0.2 to exclude non-white matter voxels. Each participant’s aligned FA map was then projected onto this skeleton, resulting in an alignment-invariant representation of the central trajectory of white matter pathways for all subjects. This process was repeated for each subject’s MD, AD and RD map using the individual registration and projection vectors obtained in the FA nonlinear registration and skeletonization. Voxel-wise differences in FA, MD, AD and RD values between ET patients and HC were tested using permutation-based inference for nonparametric statistical thresholding (FSL’s “randomize” function)^41^ and two-sample t-tests. The number of permutations was set to 5000 to allow robust statistical inference. Age, gender and total intracranial volume were entered into the analysis as confound regressors. For between-group comparisons, a family-wise error corrected threshold of p < 0.05 was selected using the randomize tool’s threshold-free cluster enhancement (TFCE) option^42^. The white matter tracts were identified using the ICBM-DTI-81 white matter labels atlas included with FSL^43,44^. In addition, significant white matter clusters were identified by their coordinates in Montreal Neurological Institute convention and by their cluster size.

The images shown in the current paper were created using the FSLview tool from FSL, by overlapping the group-averaged white matter skeleton (blue) and the results from between-group comparisons (family-wise error corrected threshold of p < 0.05) (red-yellow) onto a standard T1 Montreal Neurological Institute template.

### Correlation analyses

Correlation analysis was performed to study the relationship between neuropsychological test scores and each of the DTI measures. Cognitive domains (attention, executive function, verbal memory, visual memory, visuospatial ability, and language) were used as covariates of interest in the framework of a general linear model. Also, age, gender, total intracranial volume, disease duration, and total tremor score were entered in the design matrix throughout the analysis. Statistical analysis was performed using the FSL Randomize Tool with 5000 permutations. TFCE was performed to enhance cluster-like structures. In correlation analyses, there were not any clusters or voxels with analysis corrected for multiple comparisons, so the correlation results with p < 0.01 (uncorrected for multiple comparisons) were reported as statistically significant.

### Sample size and statistical analyses of clinical and neuropsychological data

In several recent publications on suitable sample sizes for DTI studies, using tract-based spatial statistics, it has been reported that a group size of approximately 20 is sufficient^25,45^.

Statistical analyses for the clinical and neuropsychological measures were conducted using Statistical Package for the Social Sciences (SPSS) Version 22.0 (SPSS, IBM Corporation). Mean scores (age and neuropsychological variables) were compared using two independent sample t-tests for continuous and normally distributed data, and Mann–Whitney U test for non-normally distributed data, where appropriate. The χ2 test was used to analyze differences in sex distribution.

## Results

### Clinical and Neuropsychological testing results

As this study was nested within the NEUROTREMOR project (http://www.neuralrehabilitation.org/projects/neurotremor/), a project whose main aim was to validate technically, functionally and clinically, a novel system for understanding, providing diagnostic support, and remotely managing tremors, most the ET patients who were eligible refused to participate because of lack of time because the study would have required that they come to the hospital several times duringthe study for the performance of clinical, neurophysiological (magneto-electroencephalography and electromyography recordings), neuropsychological, and imaging evaluations. Given this constraint, of the 300 ET patients seen at outpatient neurology clinics of the University Hospital “12 de Octubre” in Madrid (Spain) from October 2012 to July 2013, only 47 were eligible for the study. Of these 47 ET patients who were eligible for the study, 26 had complete neuropsychological testing (see above) and an MRI procedure with TBSS data. Of these 26 ET patients, two had dystonic features upon review of their videotapes; therefore, these two were excluded. One was excluded from the final analyses because he developed incident Parkinson’s disease during follow-up. None of the patients and controls were excluded because of neurological comorbidities or structural abnormalities on conventional MRI images.

According to Fazekas visual rating scale, all participants had a Fazekas score ≤ 1 (i.e., normal in the elderly)^46^. On the other hand, a strict criterion for head movement assessment was adopted (maximal absolute head movement less than 1.0  mm and 1.0° in the x, y, and z directions). Neither patients nor HCs were excluded from the analysis due to this criterion.

The final sample included 23 right-handed ET patients (12 women and 11 men) and 23 right-handed HC (12 women and 10 men). The 23 ET patients did not differ to a significant degree from the 23 controls in terms of age, sex, and educational level (Table 1). The mean tremor duration was 22.9 ± 16.5 years and the mean tremor rating scale score was 30.1 ± 15.0 (Table 1).

**Table 1:** Comparison of demographic, clinical and cognitive domains of essential tremor patients vs. healthy controls.

	Essential tremor patients (N = 23)	Controls (N = 23)	P value
**Age in years**	63.3 ± 13.4	61.1 ± 13.1	0.566^a^
**Number of female participants (%)**	13 (56.5%)	12 (52.2%)	0.767^c^
**Years of education**	8.0 ± 3.8	9.9 ± 3.9	0.101^a^
**Tremor duration, years**	22.9 ± 16.5	—	
**Number of patients with head tremor (%)**	7 (30.4%)	—	
**Number of patients with voice tremor (%)**	4 (17.4%)	—	
**Fahn-Tolosa-Marin tremor rating scale score**	30.1 ± 15.0	—	
**Cognitive domains**			
***Attention***			
Direct Digit Span subtest from the WAIS-III	5.6 ± 1.4	5.9 ± 1.3	0.457^a^
Coding-Digit Symbol subtest from the WAIS-III	33.0 ± 17.4	53.3 ± 19.4	0.001^a^
***Executive function***			
Stroop Color–Word Trial	26.4 ± 13.3	33.4 ± 12.1	0.074^a^
Frontal Assessment Battery	15.4 ± 2.9	16.8 ± 1.0	0.041^b^
WAIS-III Similarities subtest	16.2 ± 6.3	18.2 ± 5.6	0.264^a^
Indirect Digit Span test from the WAIS-III	3.8 ± 1.2	4.3 ± 1.1	0.158^a^
Controlled Oral Word Association Test	26.8 ± 13.6	37.0 ± 13.0	0.012^a^
***Verbal memory***			
WMS-III Word List			
*Learning list*	28.3 ± 5.6	29.0 ± 6.4	0.698^a^
*Immediate recall*	6.3 ± 2.4	6.9 ± 2.3	0.386^a^
*Delayed recall*	5.5 ± 2.6	6.8 ± 2.3	0.082^a^
*Recognition*	21.7 ± 2.1	22.3 ± 1.4	0.395^b^
***Visual memory***			
Brief Visuospatial Memory Test-Revised			
*Learning total*	23.0 ± 9.6	27.6 ± 6.7	0.073^a^
*Delayed free recall trial*	8.5 ± 3.6	10.1 ± 2.4	0.181^b^
*Recognition trial*	11.4 ± 0.9	11.8 ± 0.5	0.114^b^
***Visuospatial ability***			
Benton Judgment of Line Orientation Test	9.5 ± 2.7	10.1 ± 3.1	0.514^a^
Hooper Visual Organization Test	35.8 ± 9.4	40.9 ± 8.7	0.063^a^
***Language***			
Boston Naming Test	44.7 ± 11.7	52.6 ± 5.2	0.006^a^
Total number of animals as possible in one minute	18.7 ± 8.4	21.5 ± 6.8	0.222^a^
***Depressive symptoms***			
*17-item Hamilton Depression Rating Scale total score*	6.8 ± 5.0	5.8 ± 5.0	0.541^a^

Mean ± SD (median) and frequency (%) are reported. ^a^Student’s t tests or ^b^Mann-Whitney U test were used for comparisons of continuous data, and ^c^X2 test sex.

WAIS-III = Wechsler Adult Intelligence Scale-Third Edition.

WMS-III = Wechsler Memory Scale-Third Edition.

The results of neuropsychological testing are shown in Table 1. In several domains, ET patients’ cognitive performance was significantly worse than that of the HC. These differences involved selected tests of attention, executive function, and language.

### Comparison of DTI metrics between ET and HCs

**Figure 1:**
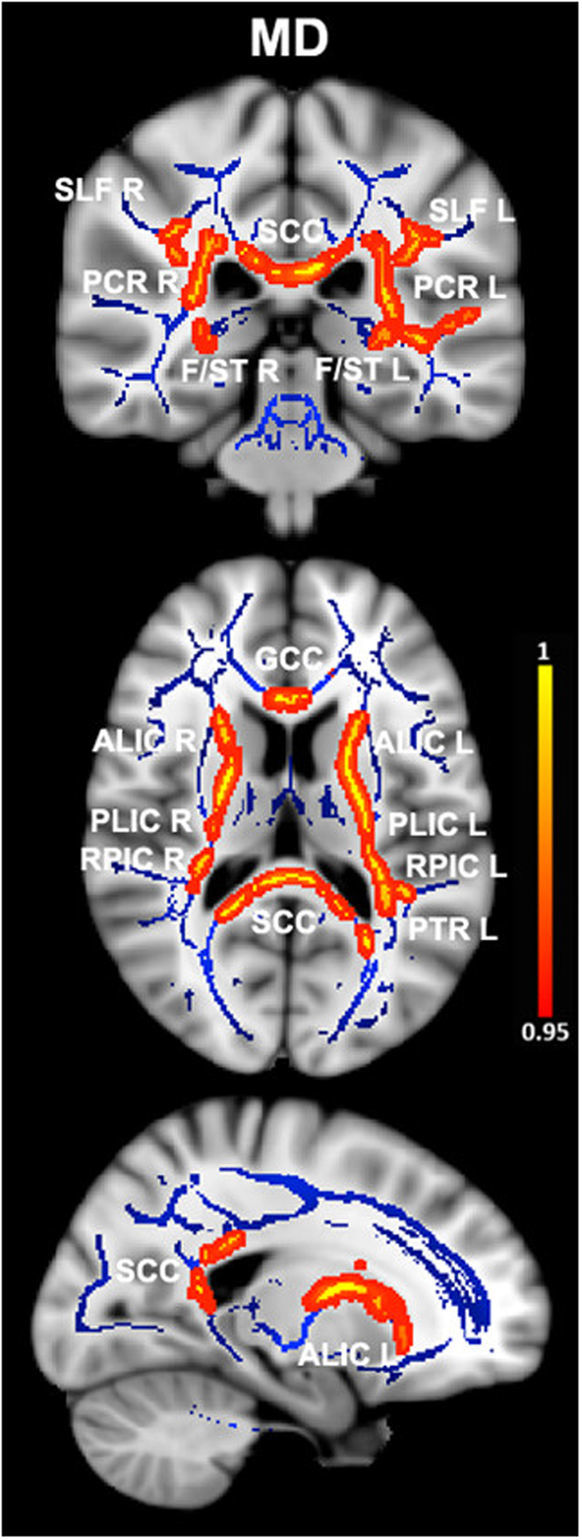
Differences in mean diffusivity values in essential tremor (ET) patients vs. healthy controls. Results from between-group comparison showing clusters with significantly increased mean diffusivity values in ET patients compared with healthy controls (family-wise error–corrected p < 0.05, red-yellow). The group-averaged white matter skeleton (fractional anisotropy threshold > 0.2) is shown in blue. Group differences are mapped onto a standard T1 Montreal Neurological Institute template. Images are in radiological convention (i.e., findings in the left hemisphere are displayed on the right and vice versa). The color bar represents statistical significance (1 minus p-value). SLF L/R, left and right superior longitudinal fasciculus; PCR L/R, left and right posterior corona radiata; SCC, splenium of the corpus callosum; F/ST L/R, left and right fornix (cres)/stria terminalis; GCC, genu of the corpus callosum; ALIC L/R, left and right anterior limb of internal capsule; PLIC L/R, left and right posterior limb of the internal capsule; RPIC L/R, left and right retrolenticular region of internal capsule; PTR L, left posterior thalamic radiation.

**Figure 2:**
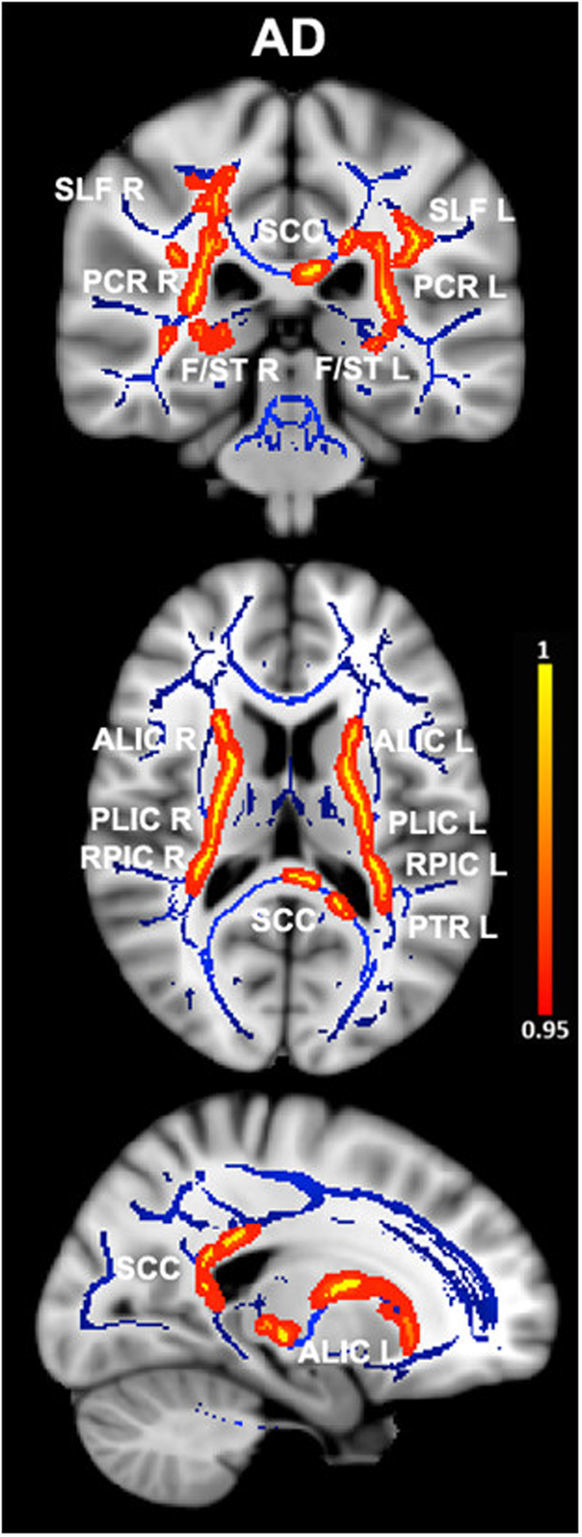
Differences in axial diffusivity values in essential tremor (ET) patients vs. healthy controls. Results from between-group comparison showing clusters with significantly increased axial diffusivity values in ET patients compared with healthy controls (family-wise error–corrected p < 0.05, red-yellow). The group-averaged white matter skeleton (fractional anisotropy threshold > 0.2) is shown in blue. Group differences are mapped onto a standard T1 Montreal Neurological Institute template. Images are in radiological convention (i.e., findings in the left hemisphere are displayed on the right and vice versa). The color bar represents statistical significance (1 minus P-value). SLF L/R, left and right superior longitudinal fasciculus; PCR L/R, left and right posterior corona radiata; SCC, splenium of the corpus callosum; F/ST L/R, left and right fornix (cres)/stria terminalis; ALIC L/R left and right anterior limb of internal capsule; PLIC L/R, left and right posterior limb of the internal capsule; RPIC L/R left and right retrolenticular region of internal capsule; PTR L, left posterior thalamic radiation.

Patients with ET demonstrated increases in MD in the bilateral posterior corona radiata, bilateral superior longitudinal fasciculus, bilateral fornix (cres)/stria terminalis, genu and splenium of the corpus callosum, bilateral anterior and posterior limbs of internal capsule, bilateral retrolenticular region of internal capsule, and left posterior thalamic radiation. See Fig. 1 and Table 2 for more details. Interestingly, with the exception of the genu of the corpus callosum, AD values were also significantly increased in the same tracts in ET patients compared with HCs (Fig. 2 and Table 3). We did not find any tract in which there was statistically significant increase of MD and AD values in HCs with respect to the ET patients. Finally, no significant differences in FA and RD were detected between groups at the P _**family-wise error-corrected**_ < 0.05 level.

**Table 2:** White matter clusters showing increased mean diffusivity in essential tremor vs. healthy control subjects.

	Montreal Neurological Institute coordinates			Cluster size (number of voxels)	P _family-wise error-corrected_
	x	y	z		
Right posterior corona radiata	31	−41	19	657	0.047
Left posterior corona radiata	−29	−40	19	458	0.046
Right superior longitudinal fasciculus	32	−3	20	954	0.047
Left superior longitudinal fasciculus	−32	−32	25	480	0.048
Right fornix (cres)/stria terminalis	29	−25	−8	160	0.047
Left fornix (cres)/stria terminalis	−30	−23	−7	136	0.046
Genu of the corpus callosum	−11	29	−6	848	0.047
Splenium of the corpus callosum	−18	−46	7	3037	0.042
Right anterior limb of internal capsule	22	22	0	750	0.047
Left anterior limb of internal capsule	−15	15	−4	1162	0.046
Right posterior limb of the internal capsule	25	−17	1	642	0.047
Left posterior limb of the internal capsule	−17	−3	7	787	0.046
Right retrolenticular region of internal capsule	31	−33	11	165	0.047
Left retrolenticular region of internal capsule	−38	−28	−3	972	0.046
Left posterior thalamic radiation	−38	−39	2	356	0.046

White matter clusters with significantly increased mean diffusivity (P _**family-wise error-corrected**_ < 0.05) in essential tremor vs. healthy control subjects. Coordinates indicate the location of the cluster peak in Montreal Neurological Institute convention.

**Table 3:** White matter clusters showing increased axial diffusivity in essential tremor vs. healthy control subjects.

	Montreal Neurological Institute coordinates			Cluster size (number of voxels)	P _family-wise error-corrected_
	x	y	z		
Right posterior corona radiata	31	−41	19	749	0.017
Left posterior corona radiata	−28	−38	19	586	0.027
Right superior longitudinal fasciculus	33	−12	23	827	0.017
Left superior longitudinal fasciculus	−34	−16	25	783	0.027
Right fornix (cres)/stria terminalis	29	−25	−8	111	0.017
Left fornix (cres)/stria terminalis	−32	−25	−6	58	0.043
Splenium of the corpus callosum	−9	−38	8	1074	0.045
Right anterior limb of internal capsule	21	21	0	812	0.017
Left anterior limb of internal capsule	−15	14	−2	708	0.027
Right posterior limb of the internal capsule	23	−20	−3	1254	0.017
Left posterior limb of the internal capsule	−24	−18	8	822	0.027
Right retrolenticular region of internal capsule	38	−26	−3	738	0.017
Left retrolenticular region of internal capsule	−33	−36	7	555	0.043
Left posterior thalamic radiation	−32	−39	11	76	0.027

White matter clusters with significantly increased axial diffusivity (P _**family-wise error-corrected**_ < 0.05) in essential tremor vs. healthy control subjects. Coordinates indicate the location of the cluster peak in Montreal Neurological Institute convention.

### Correlation Analyses

The following correlations (uncorrected for multiple comparisons, P < 0.01) between diffusion measures statistically different at a P _**family-wise error-corrected**_ < 0.05 level and cognitive domains scores were observed in the ET group:

1. The language domain showed a negative correlation with MD of the right superior cerebellar peduncle (P = 0.004), left corticospinal tract (P = 0.004), right cerebral peduncle (P = 0.004), left cerebral peduncle (P = 0.004), and the splenium of corpus callosum (P = 0.005), and with AD of the right parahippocampal gyrus (P = 0.001), right cerebral peduncle (P = 0.005), left cerebral peduncle (P = 0.004), left corticospinal tract (P = 0.002), and the splenium of corpus callosum (P = 0.009).
2. The verbal memory domain showed a negative correlation with MD of the right parahippocampal gyrus (P = 0.004), left parahippocampal gyrus (P = 0.002), left posterior thalamic radiation (P = 0.007), left corticospinal tract (P = 0.006), left cerebral peduncle(P = 0.008), and the splenium of corpus callosum (P = 0.007), and with AD of the left corticospinal tract (P = 0.001), and the left parahippocampal gyrus (P = 0.001).
3. The visuospatial ability domain showed a positive correlation with MD of the right parahippocampal gyrus (P = 0.004) and the left parahippocampal gyrus (P = 0.003), and with AD of the right sagittal stratum (P = 0.008), body of corpus callosum (P = 0.006) and the splenium of corpus callosum (P = 0.007).

There was no statistically significant correlation between FA and any of the cognitive domains scores or clinical scores (i.e., disease duration and total tremor score) in the ET group.

## Discussion

The present exploratory study used TBSS to analyze whole‐brain white matter microstructure in non-demented ET patients, finding increased MD in several regions including the bilateral posterior corona radiata, bilateral superior longitudinal fasciculus, bilateral fornix (cres)/stria terminalis, genu and splenium of the corpus callosum, both internal capsules, and left posterior thalamic radiation. AD was also increased in the majority of the same tracts, suggesting tract degeneration in these regions^45^. To date, only a few studies investigating white matter changes in ET patients have used the whole‐brain TBSS approach^25,27,33^. Our findings are in agreement with these previous TBSS studies^25,27,33^.

Furthermore, increased MD and AD values in different white matter areas (right superior cerebellar peduncle, left corticospinal tract, cerebral peduncles, the splenium of corpus callosum, parahippocampal gyri, and the left posterior thalamic radiation) was negatively correlated with performance on language and verbal memory (in other words, the highest AD and MD values – i.e., more white matter changes -, the worse cognitive performance). On the other hand, increased MD (in parahippocampal gyri), and AD values (in the right sagittal stratum, body of corpus callosum and the splenium of corpus callosum) was positively correlated with performance on visuospatial ability (indeed, the highest AD and MD values – i.e., more white matter changes -, the better cognitive performance). The exact mechanism linking white matter changes with specific cognitive domains in ET remains unclear. In the specific case of the positive correlations between increased MD and AD values in different white matter areas with visuospatial ability may reflect compensatory reorganization of neural circuits indicative of adaptive or extended neuroplasticity, thereby allowing ET patients to maintain the same level of cognitive performance (visuospatial ability) as HCs (see Table 1). However, further work is necessary in order to confirm this.

We recognize that correlation and causality are not the same. Demonstrating that the white matter changes in several regions preceded the cognitive changes would be an important step in establishing causality; however, in the absence of a prospective, longitudinal study, this is not possible. Nonetheless, it is more biologically plausible that the white matter changes resulted in the cognitive changes than viceversa. These regions (the superior cerebellar peduncle, left corticospinal tract, cerebral peduncles, corpus callosum, parahippocampal cortex, posterior thalamic radiation, and sagittal stratum) have been associated with cognition in some way. First, dysfunction of the frontal–thalamic–cerebellar circuitry is thought to be associated with subtle cognitive abnormalities in ET patients^9–15^. Since the superior cerebellar peduncle is involved in neural connectivity in the frontal–thalamic–cerebellar circuitry, subtle disruption of the superior cerebellar peduncle may be involved in the neural circuit deficits associated with ET. Second, the corticospinal tract consists of major efferent projection fibers that connect the motor cortex to the brain stem and spinal cord^47^. These fibers converge in the corona radiata and continue through the posterior limb of the internal capsule to the cerebral peduncle on their way to the lateral funiculus^47^. Although the corticospinal tract arises primarily from the primary motor cortex, projections from other areas including the somatosensory, cingulate, and insular cortices are also represented^48^. Thus, the corticospinal tract is likely involved in a variety of functions, including cognition. Third, the genu of the corpus callosum connects several areas of the default-mode cortical network, a structure involved in cognitive processes^49^. Previous research by our group and others has reported abnormal functioning of default mode network regions in ET^19,20^. Likewise, the splenium of the corpus callosum is rostral to a key default-mode cortical network hub, the posterior cingulate cortex^49^. Increased MD in the splenium of the corpus callosum has correlated with posterior cingulate cortex functional connectivity^50^. Second, the parahippocampal cortex, which has been associated with many cognitive processes, including visuospatial processing and episodic memory^51^, links the default-mode cortical network with the medial temporal lobe memory system^52^. Third, the posterior thalamic radiation might be also involved in intellectual performance^53^. In fact, thalamocortical circuit integrity has been found to differentiate individuals at high risk of developing Alzheimer’s disease from healthy elderly subjects, supporting the hypothesis that neurodegenerative mechanisms are active years before the patient is clinically diagnosed with dementia^54^. Finally, decreased FA has been reported in the inferior longitudinal fasciculus, a component of the sagittal stratum, in patients with mild cognitive impairment^55^. Hence, changes of these whiter matter areas and its correlation with cognitive domains in our study extends prior findings and suggests early involvement of the same in ET patients prior to the development of dementia^18^.

The study was not without limitations. First, the sample size was relatively small. However, we could detect significant differences between ET patients and HC at stringent thresholds even with these smaller numbers. Notwithstanding, it would be important to replicate these findings in a larger sample. Second, the diagnosis of ET was based on clinical criteria and further supported by normal [(123) I]FP-CIT single photon emission computed tomography scan results. None of the ET patients had post-mortem assessments, so that it was not possible to determine whether they had the types of changes that have been reported in ET^56^. Finally, the results of our correlation analyses should be interpreted carefully because they were not controlled for multiple comparisons, which may lead to false-positives.

In closing, our findings indicate that ET patients had white matter changes mainly in the corona radiata, internal capsule, corpus callosum, and superior longitudinal fasciculus. Although the correlations between cognitive performance and diffusivity in distinct white matter areas suggests that white matter changes might be involved in the pathogenesis of cognitive deficits in ET, our findings should be considered only as a preliminary result, more for exploratory purposes than for a solid conclusion. Further additional studies with larger samples are required.
