# Author Correction: White matter microstructural changes are related to cognitive dysfunction in essential tremor

**DOI:** 10.1038/s41598-018-22516-1

**Published:** 2018-03-06

**Authors:** Julián Benito-León, Virginia Mato-Abad, Elan D. Louis, Juan Antonio Hernández-Tamames, Juan Álvarez-Linera, Félix Bermejo-Pareja, Ángela Domingo-Santos, Luis Collado, Juan Pablo Romero

**Affiliations:** 1 Department of Neurology, University Hospital “12 de Octubre”, Madrid, Spain; 2 Centro de Investigación Biomédica en Red sobre Enfermedades Neurodegenerativas (CIBERNED), Madrid, Spain; 3 Department of Medicine, Faculty of Medicine, Complutense University, Madrid, Spain; 4 Neuroimaging Laboratory, Center for Biomedical Technology, Rey Juan Carlos University, Móstoles, Madrid, Spain; 5 Department of Neurology, Yale School of Medicine, Yale University, New Haven, CT USA; 6 Department of Chronic Disease Epidemiology, Yale School of Public Health, Yale University, New Haven, CT USA; 7 Center for Neuroepidemiology and Clinical Neurological Research, Yale School of Medicine, Yale University, New Haven, CT USA; 8 Department of Radiology, Hospital Ruber International, Madrid, Spain; 9 Faculty of Biosanitary Sciences, Francisco de Vitoria University, Pozuelo de Alarcón, Madrid, Spain; 10 Brain Damage Service, Hospital Beata Maria Ana, Madrid, Spain; 11 Clinical Research Unit, University Hospital “12 de Octubre”, Madrid, Spain

Correction to: *Scientific Reports* 10.1038/s41598-017-02596-1, published online 07 June 2017

In this Article, the author contributions section is incomplete:

“Dr. Benito-León (jbenitol67@gmail.com) collaborated in: (1) the conception, organization and execution of the research project; (2) the statistical analysis design, and; (3) and the writing of the manuscript first draft and the review and critique of the manuscript. Dr. Mato-Abad (virginia.mato@urjc.es) collaborated in: (1) the conception, organization of the research project; (2) the statistical analysis design; and (3) the review and critique of the manuscript. Dr. Louis (elan.louis@yale.edu) collaborated in: (1) the conception, organization of the research project; and (2) the review and critique of the manuscript. Dr. Hernández-Tamames (juanantonio.hernandez@ctb.upm.es) collaborated in: (1) the conception, organization of the research project; and (2) the review and critique of the manuscript. Dr. Álvarez-Linera (jalinera@ruberinternacional.es) collaborated in: (1) the conception, organization of the research project; and (2) the review and critique of the manuscript. Dr. Domingo-Santos (gela_yo@hotmail.com) collaborated in: (1) the conception, organization of the research project; and (2) the review and critique of the manuscript. Dr. Luis Collado (lcollado@med.ucm.es) in: (1) the review and critique of the manuscript. Dr. Romero (juanpa5@hotmail.com) collaborated in: (1) the conception, organization of the research project; and (2) the review and critique of the manuscript.”

should read:

“Dr. Benito-León (jbenitol67@gmail.com) collaborated in: (1) the conception, organization and execution of the research project; (2) the statistical analysis design, and; (3) and the writing of the manuscript first draft and the review and critique of the manuscript. Dr. Mato-Abad (virginia.mato@urjc.es) collaborated in: (1) the conception, organization of the research project; (2) the statistical analysis design; and (3) the review and critique of the manuscript. Dr. Louis (elan.louis@yale.edu) collaborated in: (1) the conception, organization of the research project; and (2) the review and critique of the manuscript. Dr. Hernández-Tamames (juanantonio.hernandez@ctb.upm.es) collaborated in: (1) the conception, organization of the research project; and (2) the review and critique of the manuscript. Dr. Álvarez-Linera (jalinera@ruberinternacional.es) collaborated in: (1) the conception, organization of the research project; and (2) the review and critique of the manuscript. Dr. Bermejo-Pareja (fbermejop2013@yahoo.es) collaborated in: (1) the conception, organization of the research project; and (2) the review and critique of the manuscript. Dr. Domingo-Santos (gela_yo@hotmail.com) collaborated in: (1) the conception, organization of the research project; and (2) the review and critique of the manuscript. Dr. Luis Collado (lcollado@med.ucm.es) in: (1) the review and critique of the manuscript. Dr. Romero (juanpa5@hotmail.com) collaborated in: (1) the conception, organization of the research project; and (2) the review and critique of the manuscript.”

